# The Association of Sex with Unplanned Cardiac Readmissions following Percutaneous Coronary Intervention in Australia: Results from a Multicentre Outcomes Registry (GenesisCare Cardiovascular Outcomes Registry)

**DOI:** 10.3390/jcm11226866

**Published:** 2022-11-21

**Authors:** Andre Conradie, John Atherton, Enayet Chowdhury, MyNgan Duong, Nisha Schwarz, Stephen Worthley, David Eccleston

**Affiliations:** 1 Friendly Society Private Hospital, Bundaberg, QLD 4670, Australia; 2 Royal Brisbane and Women’s Hospital, Brisbane, QLD 4029, Australia; 3 GenesisCare, Leabrook, SA 5068, Australia; 4 North Shore Cardiology, St Leonards, NSW 2065, Australia; 5 Melbourne Private Hospital, Parkville, VIC 3052, Australia

**Keywords:** acute coronary syndrome, PCI, sex, unplanned readmissions

## Abstract

Background and aim: Unplanned cardiac readmissions in patients with percutaneous intervention (PCI) is very common and is seen as a quality indicator of in-hospital care. Most studies have reported on the 30-day cardiac readmission rates, with very limited information being available on 1-year readmission rates and their association with mortality. The aim of this study was to investigate the impact of biological sex at 1-year post-PCI on unplanned cardiac readmissions. Methods and results: Patients enrolled into the GenesisCare Cardiovascular Outcomes Registry (GCOR-PCI) from December 2008 to December 2020 were included in the study. A total of 13,996 patients completed 12 months of follow-up and were assessed for unplanned cardiac readmissions. All patients with unplanned cardiac readmissions in the first year of post-PCI were followed in year 2 (post-PCI) for survival status. The rate of unplanned cardiac readmissions was 10.1%. Women had a 29% higher risk of unplanned cardiac readmission (HR 1.29, 95% CI 1.11 to 1.48; *p* = 0.001), and female sex was identified as an independent predictor of unplanned cardiac readmissions. Any unplanned cardiac readmission in the first year was associated with a 2.5-fold higher risk of mortality (HR 2.50, 95% CI 1.67 to 3.75; *p* < 0.001), which was similar for men and women. Conclusion: Unplanned cardiac readmissions in the first year post-PCI was strongly associated with increased all-cause mortality. Whilst the incidence of all-cause mortality was similar between women and men, a higher incidence of unplanned cardiac readmissions was observed for women, suggesting distinct predictors of unplanned cardiac readmissions exist between women and men.

## 1. Introduction

Unplanned readmissions in patients with percutaneous intervention (PCI) is very common and widely regarded as a quality indicator of in-hospital care [1]. The rate of unplanned cardiac readmissions has been reported to be as high as 16%, with the highest rate of readmission noted in the first 30 days following PCI [2]. Most studies reported on the 30-day cardiac readmission rates post-PCI and their association with increased mortality [3]. However, limited findings are available on unplanned cardiac readmissions in the first year post-PCI, especially regarding their association with mortality and predictors of unplanned cardiac readmissions [2,4]. Predictors of unplanned cardiac readmissions in the first 30 days post-PCI appear to be more associated with the clinical presentation and procedure, including the presence of peripheral vascular disease (PVD), diabetes mellitus, and renal dysfunction, and having PCI performed for acute coronary syndromes (ACSs) [2]. In contrast to this, predictors of unplanned cardiac readmissions after 1 year were more related to the burden of atherosclerosis and progression of coronary artery disease, comprising a history of previous acute coronary syndromes, angina, and evidence of heart failure [1]. Furthermore, late unplanned cardiac readmissions also appear to be strongly associated with a higher burden of cardiac risk factors [5]. By identifying the predictors of readmission, it might be possible to develop a tailored cardiac rehabilitation program with the effect of potentially reducing unplanned cardiac readmissions, improving quality of life, and reducing the risk of death [2,6]. Furthermore, female patients have been identified to be at increased risk of unplanned cardiac readmission in the first 30 days after presenting with an acute coronary syndrome (ACS) and after having PCI performed [7]; however, whether female sex is an independent predictor for unplanned cardiac readmissions in the long term is unclear. The primary aim of this study was to investigate the association of sex with unplanned cardiac readmission within the first year post-PCI procedure, and the association of unplanned cardiac readmissions in the first year with future risk of death. The secondary aim was to identify independent predictors of unplanned cardiac readmissions within the first year post-PCI.

## 2. Method

### 2.1. Study Design

The GenesisCare Cardiovascular Outcomes Registry (GCOR-PCI) is an internally funded ongoing registry for subjects undergoing PCI at 12 Australian private hospitals. The GCOR registry was established in 2008, and extensive details of the GCOR registry have been published previously [8]. Data used for this study included 13,996 patients enrolled from December 2008 to December 2020. All the patients included in the study completed 12 months of follow-up post-PCI. This study was performed in accordance with the Declaration of Helsinki and required legislation, with ethics approval granted by the Bellberry Human Research Ethics Committee (HREC#2020-03-234).

### 2.2. Study Data Collection

Sociodemographic parameters, medical history, and management, including in-hospital investigations and procedure details, are routinely recorded. Follow-up was performed by research coordinators at discharge 1 month, 6 months, and then annually post-PCI procedure. Detailed information was collected on all readmissions, and patient survival following procedures was collected during the follow-up visits. Unplanned cardiac readmission included admission for any of the following conditions after PCI: myocardial infarction (MI), unplanned percutaneous coronary intervention (PCI)/coronary artery bypass graft surgery (CABG), major bleeding, angina, cerebrovascular accident (CVA)/stroke, heart failure, and/or arrhythmia.

### 2.3. Statistical Analyses

Descriptive analyses including percent and mean with standard deviations (±SDs) were used to summarise the distribution of sociodemographic, clinical, and procedural characteristics of the patients overall as well as patient sex and age. Student’s t-test, analysis of variance, and chi-square tests were used for unadjusted comparisons of the distributions of characteristics or risk factors within different patient subgroups. Thereafter, distribution of the first instance of unplanned cardiac readmission within the first year following procedure was assessed by sex. A Cox proportional hazard regression model was then used to explore the association of sex with unplanned cardiac readmission within the first year. This analysis was adjusted for patient baseline demographic, clinical, and procedural characteristics (i.e., age, diabetes, body mass index (BMI), hypertension, hypercholesterolemia, previous medical history of myocardial infarction (MI), peripheral vascular disease (PVD), and coronary artery bypass graft surgery (CABG), smoking, renal dysfunction, PCI presentation, and multivessel disease). The effect of unplanned cardiac readmission within the first year following PCI, compared to having no unplanned readmission, was explored for an association with future all-cause mortality in year 2 using a Cox proportional hazard regression model among all patients. This analysis was also stratified by sex. Kaplan–Meier survival curves were also plotted.

Finally, analyses were conducted to identify the possible predictors of one-year unplanned cardiac readmission. To identify the predictor, we first explored the univariate association of different clinical and procedural characteristics of patient with unplanned cardiac readmission using logistic regression analyses. Thereafter, variables with a *p*-value of <0.10 for their univariate association were included in a multiple logistic regression model to identify the factors that were strongly associated with unplanned cardiac readmission. We repeated this analysis stratified by sex. All statistical analyses were performed using Stata version 17.0 for Windows (StataCorp LP, College Station, TX, USA).

## 3. Results

### 3.1. Baseline Demographics

A total of 13 996 patients (Table 1) were eligible for the study, including 10,687 (76.4%) men and 3304 (23.6%) women. Significant differences were observed between men and women, with women on average being older than men at presentation for PCI (71.7 ± 10.1 years for women vs. 67.9 ± 10.3 years for men, *p* < 0.001). A significantly higher percentage of women were older than 75 years at presentation (39.0% women vs. 25.4% men, *p* < 0.001), and fewer women presented at younger than 55 years (6.7% women vs. 11.2% men, *p* < 0.001). Regarding risk factors and comorbidities, women had a higher rate of hyperlipidaemia (88.8% in women vs. 86% in men, *p* < 0.001) and hypertension (79.9% in women vs. 71.9% in men, *p* < 0.001), with diabetes being equally distributed between women and men. Body mass index (BMI) values were significantly lower in women (28.7 ± 5.8 in women vs. 29.0 ± 4.6 in men, *p* < 0.001). For the different categories of smoking status, a marked difference was noted between women and men, with significantly more women who had never smoked (60.8% in women vs. 40.3% in men, *p* < 0.001), and significantly fewer women who were previous smokers or current smokers. Heart failure was more common in women, either as a previous history of heart failure (6.1% in women vs. 4.9% in men, *p* = 0.008), or as a current diagnosis of heart failure (4.4% in women vs. 3.3% in men, *p* = 0.004). Despite a higher rate of heart failure, women on average had a higher left ventricular ejection fraction compared to men (58.0 ± 9.9% in women vs. 56.5 ± 10.1% in men, *p* < 0.001). The prevalence of established coronary artery disease was less observed in women, as reflected by the lower rate of previous myocardial infarction (18.6% in women vs. 23.9% in men, *p* < 0.001), previous PCI (28.6% in women vs. 34.4% in men, *p* < 0.001), and previous CABG (7.4% in women vs. 11.9% in men, *p* < 0.001). In contrast to this, a higher rate of cerebrovascular disease (CeVD) was witnessed in women (8.4% in women vs. 6.7% in men, *p* < 0.001). The presence of chronic kidney disease (CKD) was evenly distributed between women and men, although a higher percentage of women presented with stage 2, 3, and 4 CKD. Regarding the presenting diagnosis for PCI, no difference was noted between men and women.

### 3.2. Procedure Characteristics

On presenting for PCI (Table 2), women on average had fewer lesions per procedure, and presented with a significantly lower rate of multivessel disease (38.8% in women vs. 46.3% in men, *p* < 0.001). This lower rate of multivessel disease was persistently present across all three different age groups (*p* < 0.001) (Supplementary Table S2). When assessing access site, the femoral approach was more commonly employed in women (59.4% in women vs. 57.3% in men, *p* = 0.03), with a lower rate noted in women for the radial approach (40.4% in women vs. 42.4% in men, *p* = 0.04). This marked difference in vascular access was evident in both younger (<55 years) as well as older women (>75 years) (Supplementary Table S2). Women presented with a lower rate of de novo lesions (81.2% in women vs. 85.4% in men, *p* < 0.001), while chronic total occlusions (CTO) were more commonly present in men (5.1% in men vs. 3.7% in women, *p* = 0.003). When assessing complexity of lesion morphology using the ACC/AHA (American College of Cardiology/American Heart Association) classification, type B_2_/C lesions were more commonly noted in men (44.2% in men vs. 39.2% in women, *p* < 0.001). In the older group (>75 years), this difference in the complexity of lesions was to a large extent absent, with a similar distribution noted between women and men (Supplementary Table S2). No difference was noted between men and women for left main (LM) disease. A higher rate of men presented with disease in a bypass graft (2.9% in men vs. 1.5% in women, *p* < 0.001), and this was especially evident in the > 75-year-old population group (5.1% in men vs. 2.1% in women, *p* < 0.001). The rate of bifurcation lesions was very similar between men and women. The physiological assessment of severity of coronary artery stenosis (fractional flow reserve (FFR)/ flow wire) was more commonly used in women (*p* < 0.001). The use of bare-metal stents (BMSs) was similar in women and men, while the rate of drug-eluting stents (DESs) was higher in men (92.1% in men vs. 90.7% in women, *p* = 0.02). Women received on average fewer stents, shorter stents, and smaller-diameter stents than men per procedure. Procedural success was very similar for both men and women, with no differences noted for complications.

### 3.3. Clinical Events and Outcomes

**Figure 1:**
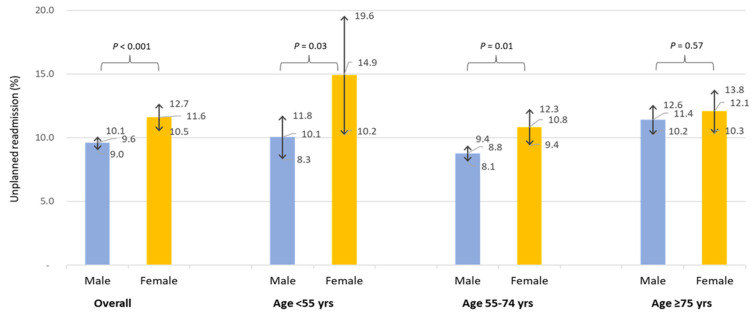
Unplanned cardiac readmission (first instance) and corresponding 95% confidence interval within first year following procedure by sex and age.

**Figure 2:**
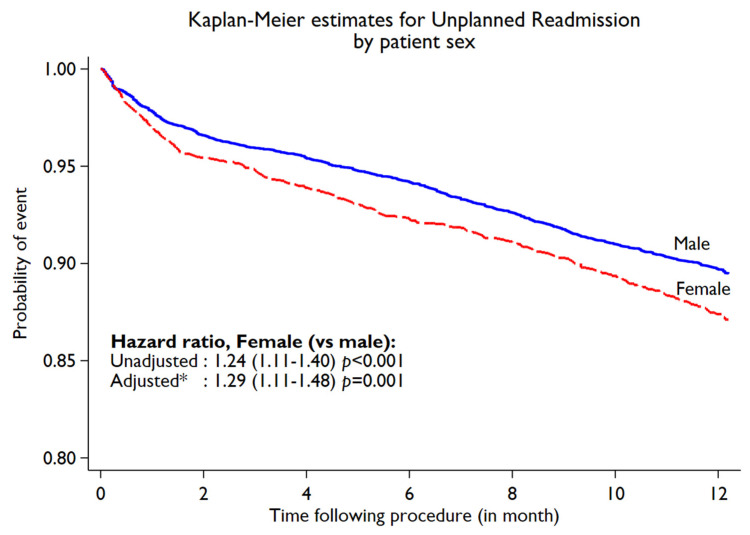
Kaplan–Meier curve for unplanned cardiac readmissions within first year following PCI by sex. * Adjusted for age, diabetes, BMI, hypertension, hypercholesterolemia, previous history of MI, PVD, or CABG, smoking, renal dysfunction, pci presentation, and multivessel disease.

**Figure 3:**
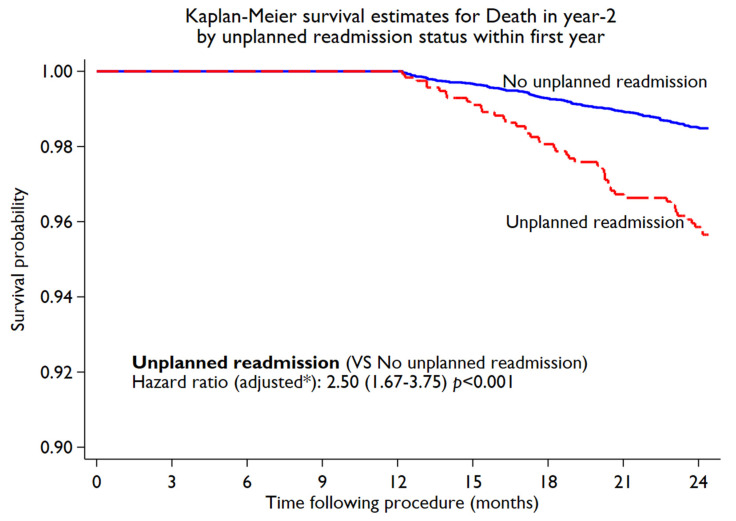
Association of unplanned cardiac readmissions in first year with death in year 2 among the study cohort. * Adjusted for age, diabetes, BMI, hypertension, hypercholesterolemia, previous history of MI, PVD, or CABG, smoking, renal dysfunction, pci presentation, and multivessel disease.

**Figure 4:**
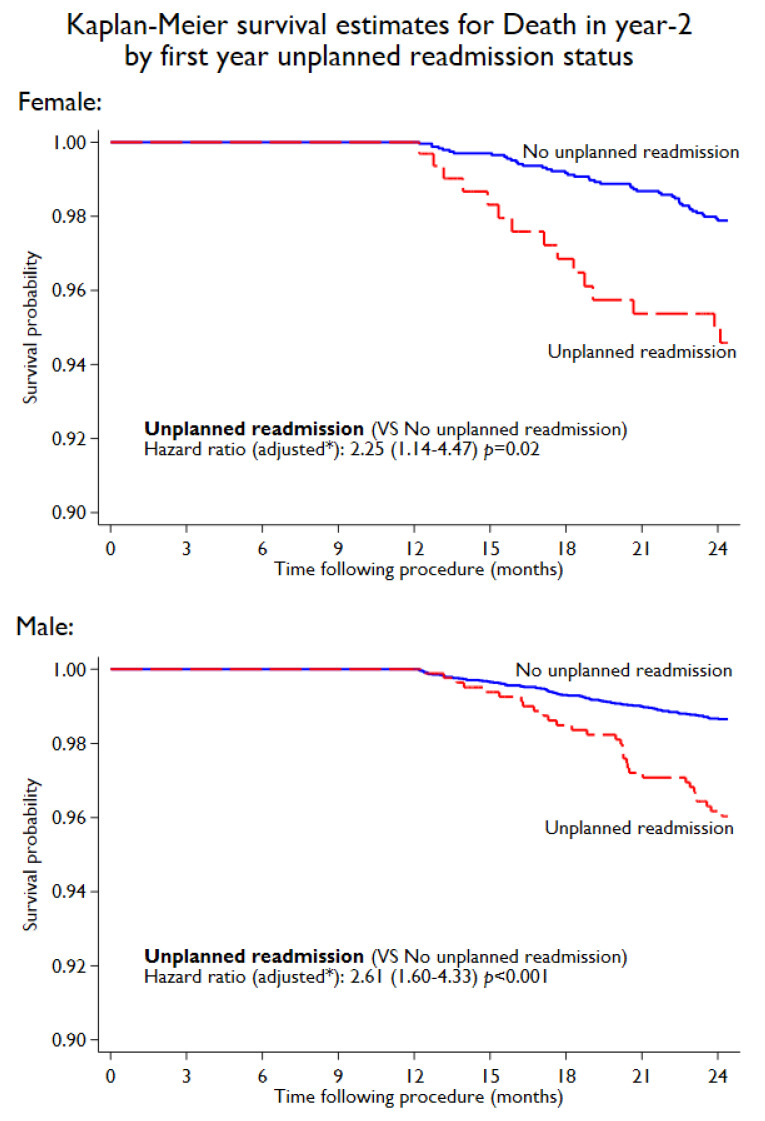
Association of unplanned cardiac readmissions in first year with death in year 2 for female and male patients. * Adjusted for age, diabetes, BMI, hypertension, hypercholesterolemia, previous history of MI, PVD, of CABG, smoking, renal dysfunction, pci presentation, and multivessel disease.

During the first year following PCI, 1408 (10.1%) unplanned cardiac readmissions (first instance) were observed. The incidence of unplanned cardiac readmissions was significantly higher in women compared to men (11.6% in women vs. 9.6% in men, *p* < 0.001). On subgroup analysis, by age, a higher rate of unplanned cardiac readmissions among women was observed (Figure 1). However, with aging, the difference in unplanned readmissions decreased. Younger women (<55 years) (Figure 1) had a significantly higher readmission rate compared to men (14.93% in women vs. 10.05% in men, *p* = 0.001), while no significant difference was noted between women and men in the ≥75-year-old age groups. The Kaplan–Meier estimated rate (Figure 2) for unplanned cardiac readmissions in women at 1 year was significantly higher compared to that for men. When adjusted for possible risk factors and confounders (Figure 2), we observed a 29% increase in unplanned readmission in women (HR 1.29, 95% CI 1.11 to 1.48; *p* = 0.001) compared to men. We further observed no difference when the population was stratified by ACS presentation among female patients (ACS: HR 1.33, 95% CI 1.08 to 1.63; *p* = 0.01. Non-ACS: HR 1.23, 95% CI 1.00 to 1.51; *p* = 0.05). We observed 186 deaths in year 2 post-PCI among 12744 patients (out of 13996) who completed the year 2 follow-up. The deaths in year 2 by unplanned cardiac readmission status in year 1, stratified by sex, are summarised in  Supplementary Table S4. Patients who experienced unplanned cardiac readmissions in the first year following PCI had a significantly increased risk of all-cause mortality (HR 2.50, 95% CI 1.67 to 3.75; *p* < 0.001) in year 2 compared to those who did not experience any unplanned cardiac readmissions in the first year (Figure 3). The risk of all-cause mortality associated with unplanned cardiac readmissions was similar for both women and men (Figure 4).

### 3.4. Predictors of Unplanned Cardiac Readmissions

Variables identified in a univariate model associated with unplanned cardiac readmissions and a *p*-value of less than 0.10 (Supplementary Table S3) were included in a multiple regression model. In this multivariate logistic regression model (Table 3) female sex was an independent predictor of unplanned readmissions (OR 1.31, 95% CI 1.07 to 1.60; *p* = 0.01). When assessing the overall cohort for independent predictors of unplanned cardiac readmissions, patients were at risk if they had a history of previous CABG and atrial fibrillation. Patients with renal dysfunction were also at increased risk of readmission with a strong trend towards significance (OR 1.40, 95% CI 1.00 to 1.96; *p* = 0.05). The stent size was also inversely related to increased cardiac readmissions. If the target vessel for PCI was the LAD artery or a bypass graft vessel, the risk of unplanned cardiac readmission was significantly increased. Treatment for in-stent stenosis was also associated with an increased risk of readmissions, with a strong trend towards significance (OR 1.49, 95% CI 1.00 to 2.23; *p* = 0.05). When testing for significance in a multivariate model stratified by sex, a significant difference was noted for the independent predictors of readmission between women and men (Table 3). Predictors of unplanned readmission in women included a history of previous CABG, atrial fibrillation, in-stent restenosis, more complex (B_2_C) lesions, and the size of the stent diameter. Independent predictors of readmission in men included atrial fibrillation, a presenting diagnosis of unstable angina, and if a graft vessel was the target vessel.

## 4. Discussion

This large, multicentre study confirmed that female patients were at higher risk of unplanned cardiac readmissions in the first year post-PCI, with a significant difference observed between men and women for predictors of unplanned cardiac readmissions. The greatest risk of unplanned cardiac readmission was observed in the younger female population (<55 years of age), with the difference observed between men and women in the older age group (>75 years) being less apparent. The risk of mortality was 2.5-fold higher in patients with unplanned cardiac readmissions, with the risk being similar for women and men.

Unplanned cardiac readmission has been identified as a very important quality metric and can be a potential indicator of quality of care, especially in the first 30 days post-PCI. The vascular approach and medication patients received during PCI may increase their risk for complications, including bleeding and renal failure [9]. These complications are often associated with an increased risk of mortality, justifying the inclusion of unplanned readmissions as a quality-of-care metric [4]. The association of readmissions with the PCI index, however, diminishes over time, and the factors involved in late cardiac readmissions most likely will be different, with multiple confounding factors implicated [2]. The rationale for this study, which is supported by the literature, was that the two most important factors implicated in late cardiac readmissions were female sex and the burden of coronary artery disease [5,10,11].

### 4.1. One-Year Unplanned Cardiac Readmission Rates

A very high readmission rate after PCI has been previously reported. The readmission rate at 6 months was 25% in the large Nationwide Readmission Database (NRD) [4], while a large Danish registry [12] reported a readmission rate of 50.4% at 1 year. In a systematic review by Kwok et al., the 1-year readmission rate for cardiac-related problems was reported to vary from 18.6% to 50.4% [13]. It was noted in both the Danish registry and the NRD that cardiac causes of readmissions peaked between 7 and 30 days, followed by a gradual decline in readmissions in both registries [4]. In the GCOR database, a 1-year readmission rate of 10.1% (*n* = 1408) was recorded. A possible explanation for the low readmission rate in this study might be that 55% of the patient population presented for PCI as an elective procedure. In the study by Hansen et al., both STEMI as well as NSTEMI were independent predictors of 1-year readmission, using stable angina as a reference [12]. It is well-described in the literature that patients presenting with acute coronary syndromes, especially NSTEMI, will have a much higher rate of comorbidities, more complex disease, and often are older, with a higher risk of complications including bleeding, heart failure and gastro-intestinal-related complications, subsequently increasing the risk of readmissions [14]. The patient demographics in the GCOR database, however, were very comparable with those in the NRD database, with only 51% of patients in the NRD database presenting with an acute coronary syndrome. The low readmission rate noted in this study was also in line with another study from the Melbourne Interventional Group registry reporting a 1-year readmission rate of 12.2% [15].

### 4.2. Difference by Sex for Unplanned Cardiac Readmissions

In most of the randomised controlled studies (RCT) on PCI and its outcome, women comprised only a small portion of the study population, and thus very limited data are available on sex-specific outcomes post-PCI [16]. In a major study assessing data from the large National Cardiovascular Data Registry (NCDR) CathPCI Registry, outcome was assessed post-PCI for differences by sex [16]. Unfortunately, rehospitalisation was not assessed as an outcome measure, but no difference was noted between men and women at 30 months post-PCI regarding mortality, bleeding, or revascularisation. When unplanned readmission was incorporated as an outcome measure, multiple observational studies confirmed that female sex was associated with a higher risk for readmission post-PCI, with one study identifying women to be at higher risk for multiple (≥3) readmissions in the 6 months post-acute coronary syndrome [17]. In this study, looking at patients in the GCOR registry, unplanned readmissions in the first-year post-PCI were significantly higher in women compared to men, with the biggest difference noted in the younger age group (<55 years). This finding was concordant with the TRIUMPH (Translational Research Investigating Underlying Disparities in Acute Myocardial Infarction Patients’ Health) registry [10,18], where younger women (<55 years) had a higher readmission rate for acute coronary syndromes but a much lower rate of readmissions associated with revascularisation. The higher readmission rate observed is unlikely to be associated with the burden of disease, with younger women presenting with less significant obstructive disease, less complex disease, and often an acute coronary syndrome with normal coronary arteries on angiography [18,19]. A possible explanation for the higher readmission rate in younger women is the presence of microvascular disease and coronary artery spasm associated with symptoms of unstable angina. In addition to this, other factors might also contribute to the higher readmission rate noted in women compared to men. When assessing the quality of care at discharge, women were less likely to receive prescriptions for evidence-based medications and anti-anginal treatment [20,21]. It was also noted in the VIRGO study (Variation in Recovery: Role of Gender on Outcomes of Young AMI Patients) that women presenting with ACS had a bigger cluster of metabolic risk factors present [19,22]. The poor management of comorbidities and risk factors, as well as lower prescription rates of anti-anginal medication, are likely significant contributors to the higher readmission rates witnessed in women. This observation was strongly supported by the TRANSLATE-ACS (Treatment With Adenosine Diphosphate Receptor Inhibitors: Longitudinal Assessment of Treatment Patterns and Events after Acute Coronary Syndrome) study, confirming a significant increase in the 1-year readmission rate to be associated with a low prescription rate of anti-anginal medication in female patients [20]. Socioeconomic factors, especially depression, also play a significant role in readmissions. In patients presenting with ACS, women had a higher prevalence of depression compared to men, with younger women being more prone to depression. A diagnosis of depression was very strongly associated with a 3-fold increase in the risk of future cardiac events and readmission [23]. The findings from this study are aligned with the literature, with female patients, especially those younger than 55 years, being at higher risk for readmission. It is therefore critical that we optimise medication prescription and address psychosocial factors to improve outcomes in female patients.

### 4.3. Unplanned Cardiac Readmissions and Association with Mortality

The association between 30-day unplanned cardiac readmissions and mortality has been extensively investigated, with multiple studies reporting a significant increase in all-cause mortality [3,24,25]. Very limited information, however, is available on 1-year unplanned cardiac readmission and its long-term association with all-cause mortality. In this study, in patients who completed the 1-year follow-up, any unplanned cardiac readmission in the first year post-PCI was associated with a 2.5-fold higher risk of mortality. A possible explanation for the increase in all-cause mortality witnessed could be the higher burden of underlying coronary artery disease and comorbidities as suggested by the predictors of unplanned cardiac readmission. Although female patients were at significantly higher risk of unplanned readmissions, mortality was very similar for both sexes.

### 4.4. Independent Predictors of Unplanned Cardiac Readmission for the Study Cohort

In the GCOR database, the predictors of readmission were identified as female sex, previous CABG, atrial fibrillation, CKD, in-stent stenosis, stent diameter, PCI for LAD artery, and coronary artery bypass graft vessels. Similar to the TRIUMPH study [10], the independent predictors of unplanned cardiac readmission were significantly different from the traditional cardiovascular risk factors.

It is evident from most of the studies looking at 30-day outcomes that female patients are at much higher risk for unplanned readmissions than men [18]. Very limited information, however, is available on the long-term readmission rate and predictors of readmission in patients not only presenting with acute coronary syndromes but also post-PCI [4], and whether sex is an independent predictor of unplanned readmission. In this study, female sex was identified as an independent predictor of unplanned cardiac readmissions at one year, with female patients having a 31% higher risk of readmissions. This is most likely not only of statistical significance, but also of clinical significance, given the narrow confidence interval.

A history of previous CABG is usually an indication of an increased burden of coronary artery disease, and has been identified in some studies as being associated with an increased risk of readmissions with acute coronary syndromes [4,5,10]. In the TRIUMPH study, a history of coronary artery bypass graft surgery was the strongest predictor for unplanned readmissions in the first year after presenting with an acute coronary syndrome (HR 2.12, 05% CI 1.45 to 3.10), followed by female sex and a history of previous PCI [10]. An interesting finding was that a higher GRACE risk score did not correlate with increased risk of readmissions, suggesting that the overall underlying burden of coronary artery disease played a more significant role in the readmission rate [10]. This was further supported by a study confirming that a history of previous coronary bypass surgery was an independent predictor of progression of disease in the non-culprit vessel, with 10% of patients receiving PCI in the 3 years post-PCI [26]. A concerning finding from this study, however, was that only 70% of these patients were still on a statin after 3 years, and only 44% were prescribed an ACEI. The findings from both the GCOR database and other studies, including the TRIUMPH [10] and REACH [27] registries, suggest that improving adherence to secondary prevention measures is very important to reduce the readmission rate.

The prevalence of atrial fibrillation in patients with coronary artery disease proceeding to PCI is very common, but varies from 5% to 22% in different studies [28,29,30,31]. Patients with atrial fibrillation undergoing PCI are not only at higher risk for in-hospital complications [31], but also for long-term adverse outcomes [32]. In the GCOR registry, the incidence of atrial fibrillation was 14.2% and was very strongly associated with the risk of readmissions in the first year post-PCI (OR 1.84, 95% CI 1.47 to 2.31; *p* < 0.001). This increased risk of readmissions could potentially be related to multiple factors. In the first instance, both atrial fibrillation and CAD are associated with multiple shared risk factors and comorbidities, including hypertension and diastolic dysfunction, increasing the risk of developing heart failure [33]. Heart failure has been identified as one of the most common cardiac causes of readmission in patients with atrial fibrillation undergoing PCI [28]. Furthermore, triple antithrombotic treatment (dual antiplatelet therapy in combination with oral anticoagulant treatment) is commonly used post-PCI for reduce the risk of ischemic events, but subsequently increases the risk of bleeding events and readmissions [30,34]. In a meta-analysis, assessing triple antithrombotic treatment (TAT) versus dual-antithrombotic treatment (DAT: single antiplatelet agent combined with oral anticoagulant), a significant reduction in bleeding was demonstrated with the use of DAT with no risk of increased thrombotic events or MACE [35]. In the PIONEER AF-PCI study [29], a significant reduction in hospital readmissions was achieved using a strategy employing DAT rather than TAT, without any effect on all-cause mortality.

The incidence of CKD was 5.7% for the whole cohort in the GCOR registry and was a significant independent predictor of unplanned cardiac readmissions (OR 1.40; 95% CI 1.00 to 1.96, *p* = 0.05). The reason for this association is not elucidated by this study, but multiple factors are potentially involved. CKD is very strongly associated with an increased risk of both bleeding and ischemic events in the first year post-PCI [36], potentially leading to increased readmissions [37]. The increased risk of ongoing ischemic events seen in patients with CKD is most likely driven by a variety of underlying pathophysiological factors, including higher platelet reactivity [38,39] and reduced bioavailability and effectiveness of thienopyridines (P_2_Y_12_ receptor antagonists) [40,41,42]. Furthermore, patients with increased severity of CKD experience more significant progression of CAD, regardless of comorbidities, and as a result are at increased risk of repeat revascularisation [37,43]. Due to the role CKD plays in increased bleeding risk in patients following PCI, CKD has been incorporated as an independent variable in different risk scores to determine high-bleeding-risk patients [36]. It is clear from this information that antithrombotic treatment in patients with CKD should be individualised to reduce the risk of bleeding and ischemic events post-PCI.

PCI for de novo bypass graft lesions was significantly associated with an increased risk of unplanned cardiac readmissions. The higher rate of readmissions associated with PCI for lesions in bypass graft vessels is most likely related to the higher complications and restenosis rate witnessed in these patients. The incidence of patients undergoing PCI for bypass grafts ranges from 5% to 18.5% [44,45,46] and is commonly associated with more significant comorbidities and an older patient population [46]. As a result of this, intervention for degenerative saphenous vein grafts (SVGs) has been associated with a higher risk of complications [46], and a high restenosis rate of 21% has been reported in patients with stenting for de novo SVG lesions [47]. It is therefore not surprising that PCI for the native coronary arteries, rather than the SVG lesions, is associated with better long-term outcomes [45].

In the pre-DES era, PCI for proximal LAD lesions was associated with an increased risk of MACE and restenosis. In two major trials with first-generation DES, PCI for the proximal LAD had very similar outcomes and was non-inferior compared to CABG [48,49]. An even further reduction in restenosis and late stent thrombosis have been demonstrated with second-generation DES [50,51], showing that restenosis and complications related to proximal LAD lesions are very similar to the treatment of lesions in other epicardial vessels [49]. Despite this, PCI for the LAD artery was still identified as an independent predictor of increased risk of unplanned cardiac readmissions in this study. The reason for this is not clear from this study and needs to be further investigated.

In the GCOR registry, treatment of in-stent restenosis with percutaneous intervention was associated with an increased risk for unplanned cardiac readmissions. Possible contributing factors are the presence of smaller vessels and the use of smaller stents (≤2.5 mm), both factors that increase the risk of recurrence of in-stent restenosis [52,53]. A very strong association between stent diameter and unplanned cardiac readmissions was noted in this study, with smaller stents putting patients at significantly higher risk of cardiac readmission. This is most likely related to a higher risk of restenosis associated with smaller lumen stents, even in the era of very low restenosis associated with second-generation DES [54].

### 4.5. Independent Predictors of Unplanned Cardiac Readmissions for Female and Male Patients

In the GCOR registry, a significant difference was noted between women and men for independent predictors of readmission. In women, a history of previous coronary artery bypass surgery and a diagnosis of atrial fibrillation were both significantly associated with unplanned cardiac readmissions. Complexity of disease (B_2_C lesions), size of stent diameters, and presenting with in-stent restenosis were also identified as independent predictors of readmission in women. In men, a presenting diagnosis of unstable angina, atrial fibrillation, and PCI for a CABG vessel were independent predictors of readmission in the first year.

A history of previous CABG was identified as a significant risk for readmissions in women with an odds ratio of 3.32 (95% CI 1.62 to 6.78, *p* = 0.001). This higher rate of readmissions noted in women could possibly be explained by multiple factors. In the Coronary Artery Surgical Study (CASS) registry, the severity of coronary artery disease by angiography was similar for women and men when presenting for CABG, but women experienced more symptoms of class III and IV angina, as well as symptoms of unstable angina not related to exercise [55]. In a large Canadian propensity-matched study looking at sex-based differences in long-term outcomes between men and women after CABG, women were older, had significantly more comorbidities, had a lower rate of previous coronary artery disease, received fewer arterial grafts, and were more likely to be incompletely revascularised [56]. It is clear from the studies available that women and men presenting with a history of CABG had a similar extent of coronary artery disease, with the biggest difference noted in women having a higher risk of incomplete revascularisation, more significant comorbidities, and being more symptomatic. The fact that women were more likely to be readmitted for unstable angina and heart failure, while the rate of myocardial infarction was similar for women and men [56], suggests that the higher rate of readmissions is most likely explained by other factors. When presenting with unstable angina/NSTEMI, a marked difference was noted in the cardiac biomarkers between men and women, with women more often presenting with positive hs-CRP and BNP levels, and men with elevated CK-MB and troponin levels [57]. Wiviott et al., proposed that the underlying mechanism in women was more likely related to microvascular disease and intravascular inflammation, while in men plaque rupture and thrombus formation were most likely involved [55,57]. This underlying difference in the pathophysiology between women and men could possibly explain the higher rate of readmissions seen in younger women in the GCOR registry as well as in other studies (e.g., the TRIUMPH study). Furthermore, in the GCOR registry, women were significantly older and had more comorbidities, suggesting that a history of previous CABG might serve as a surrogate marker for a higher burden and complexity of coronary artery disease, thereby predisposing women to an increased risk of readmissions after percutaneous interventions.

In the GCOR database, treatment of in-stent restenosis with percutaneous intervention was associated with an increased risk for unplanned readmission in women. Possible contributing factors include the presence of smaller vessels in women and women receiving smaller stents (≤ 2.5 mm), both factors that increase the risk of in-stent restenosis [52,53]. Complexity of lesions (B_2_C lesions) and the size of the stent were both significantly associated with an increased risk of unplanned cardiac readmissions in women.

Atrial fibrillation was identified as a significant predictor of unplanned cardiac readmissions in both sexes. Although atrial fibrillation is very common in both men and women with coronary artery disease, sex-specific differences have been observed between men and women. Men commonly present at a younger age with atrial fibrillation [33,58], while older women (>80 years) have a higher incidence of atrial fibrillation compared to men [59,60]. The cumulative lifetime risk of developing atrial fibrillation with age, however, was similar for men and women [61]. In patients with concomitant coronary artery disease and atrial fibrillation, more women suffer from atrial fibrillation and are notably older than men [28]. This was very similar in the GCOR registry, where women more often presented with atrial fibrillation and were significantly older compared to men. A significant difference is observed with comorbidities in men and women with atrial fibrillation, with women more often presenting with hypertension, heart failure with preserved ejection fraction (HFpEF), and cerebrovascular events [58,62]. Men more commonly presented with heart failure with reduced ejection fraction (HFrEF) and had a higher burden of coronary artery disease [58,62]. Despite the differences observed, both men and women are at increased risk of adverse outcomes, which most likely explains why both sexes were at risk of unplanned cardiac readmissions in this study. This is further underscored in a large PCI study with DES, where patients with atrial fibrillation had a significantly increased 1-year risk of bleeding and MACE [32].

Unstable angina was identified as an independent predictor for an increased rate of late readmissions in men. A possible explanation for the role unstable angina plays in readmission is the progression of disease in the non-culprit lesions [63]. It has been previously reported that, even though the non-target lesions were angiographically mild, progression of disease occurred in 11.6% of patients at 3 years, with underlying plaque burden being the most important factor [63]. This is further supported by Glaser et al., who demonstrated significant progression of disease in non-target lesions, especially in patients with evidence of a high burden of coronary artery disease. In their study, they recorded an event rate of 5.8% at 1-year post-index event [26,64]. In both the GCOR registry as well as other studies, men presented with a much higher rate of pre-existing coronary artery disease, including a history of previous CABG and PCI, and a higher rate of multivessel disease [19,55,65]. The suggestion, therefore, is that the association between unstable angina and an increased risk of readmissions in men is most likely associated with a higher burden of coronary artery disease and more extensive cases compared to female patients. This is also in line with data from the REACH (Reduction of Atherothrombosis for Continued Health) registry, showing that multivessel disease is associated with an increase in cardiovascular events and mortality, and with a higher readmission rate in men [6,27].

PCI for a bypass graft was associated with an increased risk of readmission in both men and women but did not reach significance in women, most likely due to the smaller number of women with a history of previous CABG. The underlying mechanism, increasing the risk of restenosis, is most likely similar for women and men. Very limited information, however, is available from the literature, with the majority of the studies including mostly men; hence, a direct comparison between men and women is not available [47,66].

## 5. Limitation

Limitations were evident in this study. Firstly, enrolling patients only from private hospitals in Australia might have influenced the lower cardiac readmission rates observed in this study. It is well-established that patients in private hospitals in Australia, presenting with acute coronary syndromes (ACSs), have a higher rate of PCI compared to patients in public hospitals [67]. This higher rate of revascularisation might have contributed to the lower cardiac readmission rates, but no comparative studies are available to address the differences in outcome between public and private hospitals in Australia. Secondly, patients with coronary artery disease on medical treatment and going for coronary artery bypass surgery were not included in this study. The results therefore cannot be seen as representative of all patients with coronary artery disease.

## 6. Conclusions

Unplanned cardiac readmissions in the first year post-PCI are very strongly associated with increased all-cause mortality. Factors associated with unplanned cardiac readmissions included a higher burden of underlying coronary artery disease as well as comorbidities. Female sex was strongly associated with an increased risk of cardiac readmissions, with the biggest risk identified in female patients younger than 55 years. Although significant differences were noted between women and men for independent predictors of unplanned cardiac readmissions, this likely does not explain the higher incidence of cardiac readmissions witnessed in women. Further studies are needed to address the difference in readmissions between women and men, and whether gender-based differences might be a significant contributor to adverse outcomes.

## Figures and Tables

**Table 1:** Patient Characteristics.

Factor	Total	Male	Female	*p*
N	13,996	10,687	3304	
Age (in yr), mean (SD)	68.8 (10.4) (*n* = 13979)	67.9 (10.3) (*n* = 10677)	71.7 (10.1) (*n* = 3297)	<0.001
Age categories				
<55 years	1413 (10.1%)	1192 (11.2%)	221 (6.7%)	<0.001
55–74 years	8565 (61.3%)	6770 (63.4%)	1790 (54.3%)	<0.001
>75 years	4001 (28.6%)	2715 (25.4%)	1286 (39.0%)	<0.001
Risk Factors				
Diabetes	3433 (24.7%)	2622 (24.7%)	810 (24.7%)	0.99
Hypertension	10195 (73.8%)	7582 (71.9%)	2613 (79.9%)	<0.001
Hypercholesterolemia (Chl > 5.2/on Med)	11437 (86.6%)	8681 (86.0%)	2753 (88.8%)	<0.001
Family history CAD	5065 (40.8%)	3820 (40.2%)	1245 (42.6%)	0.02
Chronic HF	710 (5.2%)	513 (4.9%)	197 (6.1%)	0.01
Current HF < 2 wks	483 (3.5%)	343 (3.3%)	140 (4.4%)	0.004
Smoking history				
Never smoked	5974 (45.1%)	4088 (40.3%)	1885 (60.8%)	<0.001
Previous smoker	6129 (46.3%)	5138 (50.7%)	988 (31.9%)	<0.001
Current smoker	1138 (8.6%)	909 (9.0%)	229 (7.4%)	0.01
BMI (kg/m^2^), mean (SD)	28.9 (4.9) (*n* = 12788)	29.0 (4.6) (*n* = 9759)	28.7 (5.8) (*n* = 3024)	<0.001
Previous MI	3100 (22.7%)	2498 (23.9%)	602 (18.6%)	<0.001
Previous PCI	4582 (33.0%)	3642 (34.4%)	938 (28.6%)	<0.001
Previous PVD	1034 (7.6%)	767 (7.4%)	267 (8.3%)	0.09
Previous CeVD	965 (7.1%)	693 (6.7%)	272 (8.4%)	<0.001
Previous CABG	1511 (10.9%)	1268 (11.9%)	243 (7.4%)	<0.001
Renal dysfunction^1^	707 (5.7%)	537 (5.7%)	170 (5.8%)	0.93
eGFR (ml/min/1.73 m^2^), mean (SD)	72.1 (17.2) (*n* = 12064)	72.9 (16.8) (*n* = 9173)	69.7 (18.3) (*n* = 2888)	<0.001
Atrial fibrillation	1556 (14.2%)	1155 (13.8%)	401 (15.7%)	0.02
Ejection fraction, mean (SD)	56.9 (10.1) (*n* = 11548)	56.5 (10.1) (*n* = 8780)	58.0 (9.9) (*n* = 2765)	<0.001
Cardiogenic Shock	44 (0.3%)	29 (0.3%)	15 (0.5%)	0.100
Clinical presentation				
Elective	7426 (55.1%)	5735 (55.6%)	1690 (53.3%)	0.02
STEMI	915 (6.8%)	715 (6.9%)	199 (6.3%)	0.20
NSTEMI	2925 (21.7%)	2197 (21.3%)	727 (22.9%)	0.05
Unstable angina	2220 (16.5%)	1664 (16.1%)	555 (17.5%)	0.07

CAD—coronary artery disease; BMI—body mass index; HFB—heart failure; PVD—peripheral vascular disease; CeVD—cerebrovascular disease; CABG—coronary artery bypass grafting; MI—myocardial infarction; STEMI—ST elevation myocardial infarction; NSTEMI—non-ST-elevated myocardial infarction; SD—standard deviation. ^1^ Renal dysfunction is defined as either (a) Sr. creatinine > 2 mg/dL and/or (b) having renal failure/receiving dialysis.

**Table 2:** Procedural characteristics by sex.

	Overall			
Variables	Total	Male	Female	*p*
N	13,996	10,687	3304	
Average number of lesions per procedure, mean (SD)	1.4 (0.6) (*n* = 13919)	1.4 (0.7) (*n* = 10638)	1.3 (0.6) (*n* = 3276)	<0.001
Disease extent multivessel	6191 (44.5%)	4916 (46.3%)	1274 (38.8%)	<0.001
Lesion access site				
Femoral	8032 (57.8%)	6083 (57.3%)	1945 (59.4%)	0.033
Radial	5830 (41.9%)	4505 (42.4%)	1324 (40.4%)	0.043
Brachial	41 (0.3%)	34 (0.3%)	7 (0.2%)	0.33
Coronary lesion				
De novo	11814 (84.4%)	9126 (85.4%)	2683 (81.2%)	<0.001
In stent restenosis	671 (4.8%)	524 (4.9%)	147 (4.4%)	0.29
Restenosis	63 (0.5%)	52 (0.5%)	11 (0.3%)	0.25
Other	156 (1.1%)	132 (1.2%)	24 (0.7%)	0.015
ACC/AHA morphology				
A	1561 (11.2%)	1174 (11.0%)	387 (11.8%)	0.22
B1	4077 (29.3%)	3110 (29.2%)	965 (29.5%)	0.81
B2_C	5992 (43.0%)	4706 (44.2%)	1283 (39.2%)	<0.001
Target vessel				
RCA	3738 (26.7%)	2867 (26.8%)	871 (26.4%)	0.60
LMCA	194 (1.4%)	155 (1.5%)	39 (1.2%)	0.25
LAD	5265 (37.6%)	4011 (37.5%)	1252 (37.9%)	0.71
LCx	2557 (18.3%)	2060 (19.3%)	496 (15.0%)	<0.001
Bypass	363 (2.6%)	315 (2.9%)	48 (1.5%)	<0.001
Total occlusion	601 (4.8%)	494 (5.1%)	106 (3.7%)	0.003
Bifurcation lesion	1260 (10.0%)	969 (9.9%)	291 (10.2%)	0.61
FFR used	1938 (13.8%)	1378 (12.9%)	560 (16.9%)	<0.001
Bare-metal stents (BMSs)	1942 (16.6%)	1474 (16.3%)	468 (17.7%)	0.078
Drug-eluting stents (DESs)	10,593 (91.8%)	8234 (92.1%)	2355 (90.7%)	0.021
Average number of stents per procedure	1.3 (0.9) (*n* = 13,919)	1.4 (0.9) (*n* = 10,638)	1.2 (0.9) (*n* = 3276)	<0.001
Stent length (mm); ±SD	19.2 (6.6) (*n* = 12,127)	19.3 (6.6) (*n* = 9392)	18.6 (6.6) (*n* = 2731)	<0.001
Stent diameter (mm); ±SD	3.0 (0.5) (*n* = 12,129)	3.0 (0.5) (*n* = 9394)	2.9 (0.4) (*n* = 2731)	<0.001
Procedural success	12,285 (96.7%)	9514 (96.7%)	2767 (96.8%)	0.69

RCA—right coronary artery; LMCA—left main coronary artery; LAD—left anterior descending artery; LCX—left circumflex artery; FFR—fractional flow reserve; SD—standard deviation.

**Table 3:** Predictor of unplanned readmission (the first) according to multivariate analysis.

	Overall				Male				Female			
Predictor Variables	Odds Ratio	L95% CI	U95% CI	*p*-Value	Odds Ratio	L95% CI	U95% CI	*p*-Value	Odds Ratio	L95% CI	U95% CI	*p*-Value
Age category												
<55 yr	Ref								Ref			
55–74 yr	0.79	0.59	1.05	0.10					0.56	0.34	0.91	0.02
75 + yr	0.75	0.55	1.04	0.07					0.50	0.29	0.83	0.01
Female	1.31	1.07	1.60	0.01								
Diabetes	0.90	0.74	1.11	0.34	0.90	0.70	1.14	0.37				
Hypertension	1.21	0.98	1.49	0.08	1.18	0.93	1.50	0.17				
Heart failure	1.13	0.81	1.57	0.47	1.07	0.72	1.59	0.75				
Ejection fraction	1.00	0.99	1.01	0.91	1.00	0.98	1.01	0.40				
Previous MI	1.02	0.81	1.29	0.84	1.11	0.86	1.45	0.42	0.75	0.51	1.12	0.16
Previous PCI	0.92	0.74	1.15	0.47	0.88	0.69	1.13	0.33	1.18	0.82	1.68	0.37
Previous PVD	1.25	0.92	1.70	0.15	1.35	0.95	1.92	0.09				
Previous CeVD	1.21	0.89	1.63	0.22	1.17	0.81	1.68	0.40	1.39	0.87	2.22	0.17
Previous CABG	1.60	1.18	2.17	0.03	1.31	0.92	1.87	0.14	1.95	1.18	3.24	0.01
Atrial fibrillation	1.84	1.47	2.29	<0.001	1.67	1.28	2.17	<0.001	2.10	1.48	2.97	<0.001
Renal dysfunction	1.40	1.00	1.96	0.05	1.36	0.92	2.02	0.12				
Clinical presentation												
STEMI	Ref				Ref							
NSTEMI	1.22	0.83	1.81	0.30	1.44	0.90	2.32	0.13				
UAP	1.38	0.92	2.06	0.12	1.73	1.06	2.83	0.03				
Elective	1.02	0.70	1.50	0.90	1.34	0.85	2.14	0.21				
Coronary lesion												
De novo	Ref				Ref				Ref			
In stent restenosis	1.49	1.00	2.23	0.05	1.22	0.75	1.99	0.43	2.60	1.46	4.61	0.001
Restenosis	2.82	0.77	10.29	0.12	3.48	0.94	12.96	0.06	3.02	0.30	30.04	0.35
Other de novo	1.42	0.56	3.61	0.46	1.23	0.40	3.78	0.72	1.28	0.26	6.38	0.77
ACC/AHA morphology												
A									Ref			
B1									0.85	0.56	1.30	0.46
B2_C									0.64	0.42	0.98	0.04
Target vessel												
RCA	Ref				Ref				Ref			
LMCA	0.81	0.38	1.73	0.59	0.87	0.38	1.98	0.74	1.77	0.58	5.37	0.31
LAD	1.24	1.00	1.53	0.048	1.12	0.87	1.44	0.38	1.18	0.84	1.65	0.34
LCx	1.18	0.92	1.51	0.20	1.14	0.86	1.52	0.36	1.13	0.74	1.73	0.57
Bypass	1.77	1.06	2.95	0.03	1.83	1.03	3.25	0.04	1.97	0.71	5.46	0.20
Multivessel (vs. No)	1.05	0.88	1.25	0.58	1.05	0.86	1.29	0.61				
DES (vs. BMS)	0.78	0.58	1.07	0.12	0.71	0.50	1.02	0.06				
Stent diameter (Per mm)	0.79	0.66	0.96	0.02	0.85	0.69	1.05	0.13	0.68	0.48	0.95	0.02

PVD—peripheral vascular disease; CeVD—cerebrovascular disease; CABG—coronary artery bypass grafting; MI—myocardial infarction; STEMI—ST elevation myocardial infarction; NSTEMI—non-ST-elevated myocardial infarction; UAP—unstable angina; RCA—right coronary artery; LMCA—left main coronary artery; LAD—left anterior descending artery; LCX—left circumflex artery; BMS—bare-metal stent; DES—drug-eluting stent; SD—standard deviation.

## Data Availability

The data underlying this article will be shared on reasonable request to the corresponding author.

## Supplementary Materials

The following supporting information can be downloaded at: https://www.mdpi.com/article/10.3390/jcm11226866/s1, Table S1: Patient Characteristics by sex and age; Table S2: Procedural characteristics by sex and age; Table S3: Univariate analysis to explore association of different baseline and procedural characteristics with unplanned cardiac readmission within first year following PCI; Table S4: Distribution of all-cause mortality (between 1 and 2 yr) by unplanned cardiac readmission status in first year following PCI.
