# Supplementary material for: The Association of Sex with Unplanned Cardiac Readmissions following Percutaneous Coronary Intervention in Australia: Results from a Multicentre Outcomes Registry (GenesisCare Cardiovascular Outcomes Registry)
Source: J Clin Med. 2022 Nov 21;11(22):6866. doi: 10.3390/jcm11226866 (PMC9692358; doi:10.3390/jcm11226866)
Supplement: Supplementary file 1 [file jcm-11-06866-s001.zip › jcm-2036741-supplementary.pdf]

Supplementary Table S1: Patient Characteristics by sex and age

	Age <55yr			Age 55-74 yrs			Age ≥75 yrs		
**Factor**	**Male**	**Female**	**p**	**Male**	**Female**	**p**	**Male**	**Female**	**p**
**N**	**1192**	**221**		**6770**	**1790**		**2715**	**1286**	
Age (in yr), mean (SD)	49.6 (4.5) (n=1192)	50.4 (4.0) (n=221)	0.015	66.0 (5.3) (n=6770)	67.3 (5.2) (n=1790)	<0.001	80.6 (4.2) (n=2715)	81.5 (4.5) (n=1286)	<0.001
**Risk Factors**									
Diabetes	214 (18.0%)	59 (26.9%)	0.002	1687 (25.1%)	440 (24.8%)	0.77	719 (26.7%)	309 (24.3%)	0.10
Hypertension	641 (54.6%)	128 (58.7%)	0.26	4779 (71.5%)	1363 (76.8%)	<0.001	2155 (80.6%)	1115 (87.8%)	<0.001
Hypercholesterolemia (Chl>5.2/on Med)	942 (83.9%)	177 (84.7%)	0.77	5578 (86.6%)	1512 (89.4%)	0.002	2152 (85.3%)	1058 (88.6%)	0.006
Family history CAD	584 (52.7%)	104 (50.7%)	0.60	2584 (42.3%)	797 (49.5%)	<0.001	646 (28.4%)	342 (31.0%)	0.13
Chronic HF	7 (0.6%)	2 (0.9%)	0.59	238 (3.6%)	64 (3.7%)	0.90	266 (10.1%)	131 (10.5%)	0.72
Current HF <2wks	9 (0.8%)	3 (1.4%)	0.36	158 (2.4%)	48 (2.8%)	0.38	174 (6.6%)	89 (7.1%)	0.57
Smoking history									
Never smoked	480 (41.4%)	82 (38.9%)	0.49	2564 (39.6%)	960 (56.5%)	<0.001	1040 (41.7%)	838 (70.7%)	<0.001
Previous smoker	433 (37.3%)	74 (35.1%)	0.53	3306 (51.1%)	589 (34.7%)	<0.001	1393 (55.9%)	325 (27.4%)	<0.001
Current smoker	247 (21.3%)	55 (26.1%)	0.12	601 (9.3%)	150 (8.8%)	0.56	61 (2.4%)	23 (1.9%)	0.34
BMI (kg/m^2), mean (SD)	30.3 (4.7) (n=1101)	30.7 (6.8) (n=203)	0.32	29.4 (4.7) (n=6197)	29.2 (5.9) (n=1646)	0.25	27.5 (4.2) (n=2452)	27.5 (5.2) (n=1169)	0.96
Previous MI	220 (18.9%)	31 (14.4%)	0.12	1516 (22.9%)	282 (16.1%)	<0.001	760 (28.7%)	289 (23.1%)	<0.001
Previous PCI	312 (26.5%)	59 (27.1%)	0.86	2294 (34.1%)	488 (27.5%)	<0.001	1035 (38.6%)	391 (30.7%)	<0.001
Previous PVD	14 (1.2%)	11 (5.1%)	<0.001	396 (6.0%)	83 (4.7%)	0.045	356 (13.5%)	173 (13.8%)	0.83
Previous CeVD	29 (2.5%)	5 (2.3%)	0.91	360 (5.5%)	117 (6.7%)	0.047	302 (11.5%)	150 (12.0%)	0.65
Previous CABG	44 (3.7%)	8 (3.7%)	0.97	704 (10.5%)	118 (6.6%)	<0.001	520 (19.3%)	117 (9.2%)	<0.001
Renal dysfunction[1]	22 (2.1%)	6 (3.1%)	0.36	243 (4.1%)	68 (4.3%)	0.75	271 (11.3%)	96 (8.2%)	0.006
Atrial fibrillation	30 (3.5%)	8 (4.9%)	0.37	598 (11.2%)	138 (9.9%)	0.15	525 (24.3%)	255 (25.8%)	0.35
Ejection fraction, mean (SD)	57.7 (9.1) (n=1006)	57.8 (9.7) (n=182)	0.87	57.2 (9.6) (n=5627)	58.9 (9.7) (n=1494)	<0.001	54.4 (11.4) (n=2138)	56.9 (10.2) (n=1082)	<0.001
Cardiogenic Shock	4 (0.3%)	1 (0.5%)	0.78	14 (0.2%)	8 (0.5%)	0.072	10 (0.4%)	6 (0.5%)	0.65
Clinical presentation									
Elective	515 (44.4%)	89 (41.2%)	0.39	3799 (58.0%)	991 (58.1%)	0.97	1416 (54.6%)	607 (48.9%)	<0.001
STEMI	133 (11.5%)	24 (11.1%)	0.88	434 (6.6%)	100 (5.9%)	0.25	146 (5.6%)	74 (6.0%)	0.68
NSTEMI	307 (26.4%)	68 (31.5%)	0.13	1282 (19.6%)	334 (19.6%)	0.99	607 (23.4%)	323 (26.0%)	0.077
Unstable angina	206 (17.7%)	35 (16.2%)	0.58	1030 (15.7%)	281 (16.5%)	0.46	426 (16.4%)	238 (19.2%)	0.035

CAD-coronary artery disease; BMI-Body mass index; HF-Heart failure; PVD-Peripheral vascular disease; CeVD-Cerebrovascular disease; CABG-Coronary artery bypass grafting; MI-Myocardial infarction;; STEMI-ST-elevation myocardial infarction; NSTEMI-Non-ST-elevated myocardial infarction; SD-Standard deviation.

[1]Renal failure/impairment is defined as either (a) Sr. Creatinine >2mg/dl and/or (b) having renal failure/receiving dialysis.

Supplementary Table S2 Procedural characteristics by sex and age

	Age <55yr			Age 55-74 yrs			Age ≥75 yrs		
**Variables**	**Male**	**Female**	**p**	**Male**	**Female**	**p**	**Male**	**Female**	**p**
**N**	**1192**	**221**		**6770**	**1790**		**2715**	**1286**	
Average lesion per procedure, mean (SD)	1.4 (0.7) (n=1191)	1.3 (0.5) (n=218)	0.006	1.4 (0.6) (n=6740)	1.3 (0.6) (n=1774)	<0.001	1.4 (0.7) (n=2697)	1.4 (0.6) (n=1277)	0.11
Disease extent multivessel	486 (41.0%)	73 (33.2%)	0.029	3022 (44.9%)	646 (36.4%)	<0.001	1404 (52.1%)	553 (43.2%)	<0.001
Lesion access site									
Femoral	692 (58.2%)	145 (65.6%)	0.039	3805 (56.5%)	1004 (56.6%)	0.94	1578 (58.6%)	795 (62.4%)	0.024
Radial	492 (41.4%)	76 (34.4%)	0.052	2907 (43.2%)	765 (43.1%)	0.98	1104 (41.0%)	477 (37.4%)	0.031
Brachial	5 (0.4%)	0 (0.0%)	0.33	19 (0.3%)	4 (0.2%)	0.68	10 (0.4%)	3 (0.2%)	0.48
Coronary Lesion									
De Novo	1083 (90.9%)	182 (82.4%)	<0.001	5814 (85.9%)	1457 (81.4%)	<0.001	2220 (81.8%)	1038 (80.7%)	0.42
In stent restenosis	49 (4.1%)	16 (7.2%)	0.041	316 (4.7%)	72 (4.0%)	0.24	159 (5.9%)	59 (4.6%)	0.099
Restenosis	10 (0.8%)	0 (0.0%)	0.17	27 (0.4%)	8 (0.4%)	0.78	15 (0.6%)	3 (0.2%)	0.16
Other	5 (0.4%)	0 (0.0%)	0.33	67 (1.0%)	13 (0.7%)	0.30	59 (2.2%)	11 (0.9%)	0.003
ACC/AHA Morphology									
A	140 (11.8%)	43 (19.7%)	0.001	804 (11.9%)	223 (12.6%)	0.46	229 (8.5%)	120 (9.4%)	0.35
B1	385 (32.3%)	63 (28.9%)	0.32	1978 (29.3%)	555 (31.3%)	0.11	743 (27.5%)	344 (26.9%)	0.69
B2_C	539 (45.3%)	72 (33.0%)	<0.001	2904 (43.1%)	636 (35.9%)	<0.001	1258 (46.6%)	574 (44.9%)	0.32
Target vessel									
RCA	318 (26.7%)	60 (27.1%)	0.88	1870 (27.6%)	474 (26.5%)	0.34	675 (24.9%)	337 (26.2%)	0.36
LMCA	6 (0.5%)	5 (2.3%)	0.006	84 (1.2%)	17 (0.9%)	0.31	65 (2.4%)	17 (1.3%)	0.025
LAD	503 (42.2%)	90 (40.7%)	0.68	2570 (38.0%)	689 (38.5%)	0.68	935 (34.4%)	470 (36.5%)	0.19
LCx	233 (19.5%)	31 (14.0%)	0.053	1273 (18.8%)	258 (14.4%)	<0.001	552 (20.3%)	205 (15.9%)	<0.001
Bypass	10 (0.8%)	0 (0.0%)	0.17	167 (2.5%)	21 (1.2%)	<0.001	138 (5.1%)	27 (2.1%)	<0.001
Total occlusion	73 (6.4%)	4 (2.0%)	0.015	302 (4.9%)	66 (4.3%)	0.33	119 (4.9%)	36 (3.2%)	0.027
Bifurcation lesion	137 (12.0%)	17 (8.6%)	0.17	608 (9.8%)	165 (10.8%)	0.28	223 (9.1%)	108 (9.7%)	0.54
FFR done	79 (6.6%)	26 (11.8%)	0.007	907 (13.4%)	331 (18.5%)	<0.001	391 (14.4%)	202 (15.7%)	0.28
Bare metal stents (BMS)	200 (18.9%)	21 (11.6%)	0.018	907 (15.8%)	225 (15.8%)	0.95	364 (16.2%)	222 (21.7%)	<0.001
Drug eluting stents (DES)	973 (92.7%)	173 (95.6%)	0.15	5281 (93.0%)	1322 (93.4%)	0.61	1972 (89.6%)	854 (85.9%)	0.003

	Age <55yr			Age 55-74 yrs			Age ≥75 yrs		
**Variables**	**Male**	**Female**	**p**	**Male**	**Female**	**p**	**Male**	**Female**	**p**
**N**	**1192**	**221**		**6770**	**1790**		**2715**	**1286**	
Average stents per procedure	1.5 (0.9) (n=1191)	1.2 (0.9) (n=218)	<0.001	1.3 (0.9) (n=6740)	1.2 (0.9) (n=1774)	<0.001	1.3 (0.9) (n=2697)	1.3 (0.9) (n=1277)	0.20
Stent length (mm); ±SD	19.8 (6.8) (n=1106)	18.5 (6.0) (n=188)	0.011	19.3 (6.7) (n=5949)	18.7 (6.7) (n=1475)	0.001	19.2 (6.6) (n=2327)	18.4 (6.5) (n=1062)	0.002
Stent diameter (mm); ±SD	3.0 (0.5) (n=1106)	2.9 (0.4) (n=188)	<0.001	3.0 (0.5) (n=5950)	2.9 (0.4) (n=1475)	<0.001	3.0 (0.5) (n=2328)	2.8 (0.5) (n=1062)	<0.001

RCA-Right coronary artery; LMCA-Left main coronary artery; LAD-Left anterior descending artery; LCX-Left circumflex artery; FFR-Fractional flow reserve; BMS-Bare metal stent; DES-Drug-eluting stent; SD-Standard deviation.

Supplementary Table S3: Univariate analysis to explore association of different baseline and procedural characteristics with unplanned cardiac readmission within first year following PCI

**Variables**	**Overall**			**Male**			**Female**		
	**OR**	**95% CI**	**P-value**	**OR**	**95% CI**	**P-value**	**OR**	**95% CI**	**P-value**
Age									
<55yr	1			1			1		
55-74y	0.83	0.69 - 1.00	0.05	0.86	0.70 - 1.06	0.15	0.69	0.46 - 1.03	0.07
75+yr	1.09	0.89 - 1.32	0.40	1.16	0.93 - 1.44	0.2	0.78	0.52 - 1.17	0.23
Female									
No	1								
Yes	1.24	1.09-1.40	0.001						
Diabetes									
No	1			1			1		
Yes	1.15	1.02 - 1.30	0.03	1.16	1.01 - 1.35	0.04	1.12	0.88 - 1.42	0.38
Hypertension									
No	1			1			1		
Yes	1.37	1.19 - 1.56	<0.001	1.38	1.19 - 1.62	<0.001	1.23	0.93 - 1.63	0.15
Hypercholesterolaemia[1]									
No	1			1			1		
Yes	1.10	0.92 - 1.30	0.29	1.07	0.88 - 1.30	0.49	1.13	0.79 - 1.62	0.5
CAD Family history									
No	1			1			1		
Yes	0.94	0.83 - 1.05	0.27	0.97	0.84 - 1.11	0.64	0.84	0.67 - 1.06	0.15
Smoking history									
Never smoked	1			1			1		
Previously smoked	1.07	0.95 - 1.20	0.26	1.1	0.96 - 1.26	0.18	1.14	0.90 - 1.45	0.26
Currently smoking	0.89	0.71 - 1.11	0.29	0.95	0.74 - 1.22	0.68	0.79	0.50 - 1.27	0.33
BMI, (per kg/m^2)	1.00	0.99 - 1.01	0.71	1	0.99 - 1.02	0.72	0.99	0.97 - 1.01	0.35
Heart Failure anytime (chronic/current)									
No	1			1			1		
Yes	1.65	1.37 - 1.99	<0.001	1.79	1.44 - 2.23	<0.001	1.31	0.92 - 1.87	0.14
LVEF	0.99	0.99 - 1.00	0.003	0.99	0.98 - 0.99	<0.001	1	0.99 - 1.01	0.74
Previous MI									

Variables	Overall			Male			Female		
	**OR**	**95% CI**	**P-value**	**OR**	**95% CI**	**P-value**	**OR**	**95% CI**	**P-value**
No	1			1			1		
Yes	1.30	1.15 - 1.48	<0.001	1.32	1.14 - 1.52	<0.001	1.33	1.03 - 1.73	0.03
Previous PCI									
No	1			1			1		
Yes	1.24	1.11 - 1.39	<0.001	1.19	1.04 - 1.36	0.01	1.46	1.17 - 1.83	<0.001
Previous PVD									
No	1			1			1		
Yes	1.51	1.25 - 1.82	<0.001	1.57	1.27 - 1.95	<0.001	1.33	0.93 - 1.90	0.12
Previous CeVD									
No	1			1			1		
Yes	1.61	1.34 - 1.95	<0.001	1.65	1.32 - 2.06	<0.001	1.48	1.05 - 2.09	0.03
Previous CABG									
No	1			1			1		
Yes	1.90	1.63 - 2.20	<0.001	1.88	1.59 - 2.23	<0.001	2.18	1.57 - 3.03	<0.001
Atrial Fibrillation									
No	1			1			1		
Yes	1.96	1.68 - 2.28	<0.001	1.94	1.62 - 2.33	<0.001	1.96	1.47 - 2.62	<0.001
Renal dysfunction[2]									
No	1			1			1		
Yes	1.46	1.17 - 1.82	<0.001	1.6	1.25 - 2.06	<0.001	1.1	0.68 - 1.76	0.7
Clinical presentation									
STEMI	1			1			1		
NSTEMI	1.42	1.09 - 1.86	0.01	1.5	1.10 - 2.05	0.01	1.2	0.73 - 1.99	0.47
UAP	1.71	1.31 - 2.24	<0.001	1.77	1.29 - 2.44	<0.001	1.52	0.91 - 2.52	0.11
Elective	1.17	0.91 - 1.50	0.23	1.24	0.92 - 1.67	0.16	0.98	0.61 - 1.58	0.93
Cardiogenic Shock									
No	1			1			1		
Yes	0.89	0.32 - 2.50	0.83	1.51	0.52 - 4.35	0.44	1	1.00 - 1.00	
Lesion access site									
Brachial	1			1			1		
Radial	1.18	0.36 - 3.83	0.78	1.46	0.35 - 6.13	0.6	0.59	0.07 - 4.96	0.63
Femoral	1.59	0.49 - 5.15	0.44	1.86	0.45 - 7.79	0.39	0.92	0.11 - 7.70	0.94
Coronary Lesion									

Variables	Overall			Male			Female		
	**OR**	**95% CI**	**P-value**	**OR**	**95% CI**	**P-value**	**OR**	**95% CI**	**P-value**
De Novo	1			1			1		
In stent restenosis	1.85	1.50 - 2.29	<0.001	1.64	1.27 - 2.11	<0.001	2.61	1.76 - 3.86	<0.001
Restenosis	1.33	0.63 - 2.80	0.45	1.26	0.54 - 2.96	0.6	1.72	0.37 - 8.01	0.49
Other DeNovo	1.66	0.90 - 3.08	0.11	1.61	0.79 - 3.27	0.19	1.94	0.54 - 6.91	0.31
Chronic lesion	1.06	0.42 - 2.69	0.90	1.27	0.50 - 3.23	0.62	1	1.00 - 1.00	
Stent thrombosis	1.02	0.31 - 3.35	0.98	0.8	0.19 - 3.41	0.77	2.59	0.27 - 24.93	0.41
ACC/AHA Morphology									
A	1			1			1		
B1	0.97	0.80 - 1.17	0.74	1.06	0.84 - 1.34	0.62	0.8	0.57 - 1.13	0.21
B2_C	0.99	0.82 - 1.19	0.89	1.16	0.93 - 1.44	0.2	0.69	0.49 - 0.96	0.03
Target vessel									
RCA_any	1			1			1		
LMCA	1.57	1.02 - 2.41	0.04	1.38	0.83 - 2.30	0.21	2.36	1.05 - 5.29	0.04
LAD_any	1.07	0.93 - 1.24	0.36	1.01	0.86 - 1.20	0.87	1.23	0.93 - 1.63	0.15
LCxany	1.22	1.03 - 1.45	0.02	1.21	1.00 - 1.47	0.05	1.3	0.92 - 1.84	0.14
Bypass	2.51	1.90 - 3.33	<0.001	2.42	1.78 - 3.31	<0.001	3.39	1.73 - 6.65	<0.001
Total occlusion									
No	1			1			1		
Yes	1.07	0.83 - 1.39	0.60	1.06	0.79 - 1.43	0.69	1.17	0.67 - 2.05	0.57
Disease extent multivessel									
No	1			1			1		
Yes	1.15	1.03 - 1.28	0.02	1.17	1.02 - 1.33	0.02	1.15	0.93 - 1.43	0.2
Bifurcation lesion									
No	1			1			1		
Yes	1.09	0.91 - 1.32	0.34	1.12	0.91 - 1.40	0.28	1.01	0.70 - 1.46	0.96
Stent type -BMS/DES									
BMS only	1			1			1		
DES any	0.81	0.66 - 0.99	0.04	0.78	0.61 - 1.00	0.05	0.89	0.60 - 1.32	0.58
Stent # by Procedure, mean (SD)	1.00	0.94 - 1.06	0.90	0.97	0.90 - 1.04	0.37	1.1	0.98 - 1.23	0.11
Avr Stent length, mean (SD)	0.99	0.99 - 1.00	0.27	1.00	0.98 - 1.01	0.37	1.00	0.98 - 1.02	0.79
Avr Stent diameter, mean (SD)	0.82	0.73 - 0.93	0.00	0.88	0.76 - 1.01	0.07	0.75	0.58 - 0.98	0.04

CAD-coronary artery disease; BMI-Body mass index; HF-Heart failure; LVEF-Left ventricular ejection fraction; PVD-Peripheral vascular disease; CeVD-Cerebrovascular disease; CABG-Coronary artery bypass grafting; MI-Myocardial infarction; eGFR-estimated Glomerular Filtration Rate; IABP-Intra-aortic balloon pump therapy; STEMI-ST-elevation myocardial infarction; NSTEMI-Non-ST-elevated myocardial infarction; UAP-Unstable angina; RCA-Right coronary artery; LMCA-Left main coronary artery; LAD-Left anterior descending artery; LCX-Left circumflex artery; BMS-Bare metal stent; DES-Drug-eluting stent; SD-Standard deviation.SD-Standard deviation.

[1]Hypercholesterolaemia is defined as either (a) Cholesterol level >5.2 and/or (b) Receiving medication

[2]Renal failure/impairment is defined as either (a) Sr. Creatinine >2mg/dl and/or (b) having renal failure/receiving dialysis.

Supplementary Table S4: Distribution of all-cause mortality (between 1 and 2 yr) by unplanned cardiac readmission status in first year following PCI

	Overall	No unplanned readmission in year-1	Unplanned readmission in year-1	P-value
Overall	186/12744 (1.5%)	140/11474 (1.2%)	46/1270 (3.6%)	<0.001
Male	127/9738 (1.3%)	96/8809 (1.1%)	31/929 (3.3%)	<0.001
Female	59/3001 (2.0%)	44/2660 (1.7%)	15/341 (4.4%)	0.001
